# Editorial: Cancer neuroscience: Drug repurposing targeting the innervated niche

**DOI:** 10.3389/fphar.2023.1148706

**Published:** 2023-02-22

**Authors:** Ming-Zhu Jin, Xing-Chun Gao, Wei-Lin Jin

**Affiliations:** ^1^ Department of Obstetrics and Gynecology, Xinhua Hospital Affiliated to Shanghai Jiao Tong University School of Medicine, Shanghai, China; ^2^ Department of Immunology, School of Basic Medical Science, Xi’an Medical University, Xi’an, Shaanxi, China; ^3^ Department of Neurosurgery, Yale University, New Haven, CT, United States; ^4^ Institute of Cancer Neuroscience, Medical Frontier Innovation Research Center, The First Hospital of Lanzhou University, The First Clinical Medical College of Lanzhou University, Lanzhou, China

**Keywords:** cancer neuroscience, tumor microenevironment, innervated niche, nervous system, drug repurposing

The focus of cancer research has shifted to the tumor microenvironment (TME) in the past several decades. Previously, we summarized six hallmarks of the TME: The hypoxic niche, the immune microenvironment, the metabolism microenvironment, the acidic niche, the innervated niche, and the mechanical microenvironment (Jin and Jin, 2020), which we prefer to call specialized microenvironments. Accumulative evidence has suggested that the nervous system plays a diverse role in cancer biology, including cancer growth, cancer metastasis, stress adaptation, immune remodeling, electrical hyperactivity, seizures, and neuropathic pain (Shi et al., 2022a). Monje et al. (2020) proposed a new field called “cancer neuroscience” (Monje et al., 2020). However, research in this field is still at an early stage. Therefore, we organized this Research Topic in Frontiers in Pharmacology.

Drug repurposing targeting the TME has been a wise option and has significantly reduced the time needed to make a drug ready for clinical use. β-blockers are one of the most well-known drugs used in patients with heart disease but have been revealed to influence the tumor-nerve axis. For instance, the selective β1 blocker landiolol reduces the postoperative recurrence rate of lung cancer (Yamamoto et al., 2019). Non-selective β-blockers are positively correlated with higher overall survival in ovarian cancer patients (Watkins et al., 2015). Patients suffering from cancer might also suffer from common chronic diseases, such as hypertension, diabetes, hyperlipidemia, or heart disease. Therefore, we wondered how the drugs that they took to control their comorbidities affect tumor growth and metastasis. Additionally, cancer is associated with great pain, peripheral neuropathies, and emotional and mental alterations. For these groups of patients, we would like to know what the better option is, considering the effect of the drugs on the innervated niche.

The different forms of cell death have been hotspots in cancer research in recent years, including some newly defined cell death patterns, such as ferroptosis, panapoptosis, and paraptosis. Shi et al. provided a comprehensive review highlighting the importance of autophagy in mediating the interaction between the nervous system and cancer. Earlier, Morris et al. (2018) suggested that apoptosis, necrosis, necroptosis, parthanatos, pyroptosis, and ferroptosis play a role in neurodegenerative and neuroprogressive disorders. The death of malignant cells and non-malignant cells has been suggested to mediate intercellular communication. Intensive studies on the diverse roles that different forms of cell death play in the innervated niche might be beneficial for understanding the mechanism of the crosstalk between the nervous system and cancer.

Compared with targeting other specialized microenvironments, the advantage of drug repurposing targeting the innervated niche is that it might assist in the inhibition of tumor growth and metastasis, relieve cancer-associated pain, treat peripheral neuropathies, and improve the emotional and mental response. β-blockers, chloroquine, hydroxychloroquine, verteporfin, and natural components of curcumin affect the innervated niche or the neuron in the innervated niche by either blocking autophagic flux or inducing autophagic cell death, resulting in the suppression of tumors. Melatonin has been used to treat sleep disorders. Mechanistically, melatonin inhibits nuclear factor κB (NF-κB) activity and apoptosis. Palmer et al. carried out a randomized double-blinded placebo-controlled clinical trial to examine the effect of melatonin on pain and neuroplasticity in breast cancer patients after chemotherapy and found that melatonin uptake improves descending pain modulatory system (DPMS) function and the neuroplastic state independent of its effect on sleep quality. Chemotherapy, such as paclitaxel, vincristine, oxaliplatin, and cisplatin, can damage aspects of the nervous system, such as the dorsal root ganglion, the myelin sheath, microtubules, and terminals. Santos et al. carried out a systematic review that analyzed 22 different natural compounds and compared their nociceptive activity (mechanical allodynia, thermal allodynia, thermal hyperalgesia, or mechanical hyperalgesia). Ren et al. found that 0.3 mg/kg of ketamine administered intravenously 5 min before surgery could greatly ameliorate colorectal cancer patients’ postoperative anxiety and depression.

Cancer research emphasis has changed as progress has been made in the field. The recognition of tumor hallmarks and the shift of focus from a tumor-centric view to a tumor microenvironment-centric view has largely sped up cancer research. Cancer neuroscience integrates tumor and nervous system research. Through in-depth study, we now realize that the TME is a complicated ecosystem and has a close relationship with the whole organism. Specialized microenvironments crosstalk with each other. A single focus on a particular characteristic of the TME created a gulf between basic science and clinical practice. We believe that tumor tissue biology focusing on the tissue-specific functional characteristics of tumors and tissue specificity in cancer genes and pathways might be the key to achieving breakthroughs in cancer research and treatment.
